# Cerebral Dyspepsia

**Published:** 1883-05

**Authors:** John S. Main


### CEREBRAL DYSPEPSIA. By JOHN S. MAIN, M.D.

The author strongly insists on the purely cerebal orgin of many forms of dyspepsia, where the patient is neither over-indulgent, nor intemperate, nor addicted to hurrying over meals, nor accustomed to eat coarse or unwholesome food. The cerebral form of dyspepsia is well seen, in many cases, where a healthy man, with a good appetite suddenly receives bad news when sitting down to a meal. "But, perhaps, of all conditions acting on the brain in this manner, and through the brain on the stomach, no one thing is more injurious, or more jarring to the cerebral elements, than uncertainty, and the worry caused by the same, more particularly in preternaturally, irritable subjects. In fact, it is in connection with this same worry that the form of dyspepsia I have at present under consideration most frequently occurs. The mind, in such cases, preys upon itself; the cerebral elements seem to get jarred and out of gear; and with this condition the stomach sympathizes. But in addition to worry the habitual practice of calling into action the 'reserve fund' of the cerebrum, as already mentioned, will bring about the same consequences—namely, cerebral fatigue and exhaustion, indicated chiefly by preternatural irritability; this condition, sooner or later, telling upon the digestive organs. Having said this, it is almost unnecessary to add, that such cases are most commonly met with amongst those who are engaged in the hottest part of the 'battle of life,' or 'struggle for existence;' and, again, amongst these, chiefly those whose business or profession leads to much anxiety, uncertainty, or overstretching of the mental powers. In over-aspiring, over-ambitious natures 'hope deferred' may bring about the same results; as, according to the biblical expression 'it maketh the heart sick.' My attention was drawn to several cases of dyspepsia, connected with one or other these conditions, some time ago; and what made me more strong in my view of these cases being cerebral, and not stomachic at all in their orgin, was their obstinacy under all forms of natural treatment. Latterly, I have found that the only treatment capable of doing these cases any permanent good, is a change, in the wide sense of the term—a relaxation from business or study; and as regards medicine, not such as are meant to act on the stomach directly, but those meant to act on the cerebum. Amongst these, I have found the most useful to be the bromide of ammonium, or bromide of potassium—preferably the former—given in sufficient dose a bed-time, to secure a good night's sleep, this being often very indifferent, and so tending to complicate the case; and, combined with this, to be taken three or four times during the day, such medicines as are known to have a building up effect on the nervous system. Among these, the most useful are phosphorus, or the hypophosphites, and cod-liver oil. Arsensic and quinine are often also useful, and a generous diet is always indicated. Unless the stomach has passed into a state of disease (which it may do, if overtasked when in this weakened state), any of these medicines are generally well borne. It will be well to bear in mind, however, that if the mucous membrane of the stomach be in a state of irritation, quinine, arsenic, phosphorus, the hypophosphites, and sometimes even cod-oil, are generally inadmissible."—*British Medical Journal.*

